# Bilateral Ureteral Obstruction in Children With Ulcerative Colitis and Oliguria: When and Why?

**DOI:** 10.7759/cureus.67031

**Published:** 2024-08-16

**Authors:** Maria Florou, Christos Diamantopoulos, Kleanthis Anastasiadis, Vassileios Mouravas, Despoina Tramma, Maria Tsopozidi, Elena Kkoumourou, Vassileios Lambropoulos

**Affiliations:** 1 Department of Pediatric Surgery, Aristotle University of Thessaloniki, Papageorgiou General Hospital of Thessaloniki, Thessaloniki, GRC; 2 Department of Urology, Aristotle University of Thessaloniki, Papageorgiou General Hospital of Thessaloniki, Thessaloniki, GRC; 3 Department of Pediatric Surgery, Aristotle University of Thessaloniki, General Hospital Papageorgiou of Thessaloniki, Thessaloniki, GRC; 4 Department of Pediatrics, Aristotle University of Thessaloniki, Papageorgiou General Hospital of Thessaloniki, Thessaloniki, GRC; 5 Department of Pediatric Surgery, Aristotle University of Thessaloniki, Papageorgiou General Hosital, Thessaloniki, GRC; 6 Department of Pediatric Surgery, Asritotle University of Thessaloniki, Papageorgiou General Hospital, Thessaloniki, GRC

**Keywords:** oliguria, children, ulcerative colitis, obstruction, ureterovesical junction

## Abstract

Inflammatory bowel disease (IBD) is an inflammatory clinical entity with many extraintestinal symptoms, including urinary tract manifestations. However, the bilateral ureteral obstruction is extremely rare. We report a case of bilateral ureteral obstruction in a 12-year-old male patient with ulcerative colitis (UC). Ultrasonography in the context of sudden anuria revealed bilateral ureterovesical junction (UVJ) obstruction, and the following cystoscopy verified the presence of fragile calculi in both edematous ureteral orifices. The literature data on UC in the pediatric population are scarce. Sudden deteriorating oliguria in an UC patient may result from secondary obstructive uropathy. Immediate diagnosis and treatment are essential to prevent acute kidney injury.

## Introduction

Inflammatory bowel disease (IBD) is an inflammatory disorder mainly including Crohn's disease (CD) and ulcerative colitis (UC). Inflammatory bowel disease occurs due to a combination of environmental factors and the genetically predisposed immune response of the host [1, 2]. Approximately a pediatric onset of disease occurs in 10%-25% of the cases and the rest in the early adult life [3, 4]. Renal manifestations have been described in IBD and especially in Crohn’s disease. The data concerning urinary tract manifestations in UC in the pediatric population are scarce [1, 2]. We report a rare case of bilateral ureteral obstruction in a 12-year-old male patient with UC. An ultrasound in the context of oliguria that rapidly escalated to anuria revealed bilateral proximal ureteral and ureterovesical junction (UVJ) obstruction. To the best of our knowledge, this is the first case of bilateral obstructive uropathy concerning both the UVJs in the context of pediatric UC.

## Case presentation

A 12-year-old male patient with UC was admitted to the hospital because of diarrhea with blood. The UC diagnosis was established six months prior by colonoscopy and histopathology, along with the typical clinical manifestations of diarrhea, abdominal pain, and fever. Before admission, he was treated with mesalazine on a daily oral intake and infliximab administration every two months. The remaining personal history of the boy was clear. Concerning the family history, the patient’s father, a 43-year-old man, had also been diagnosed with UC at the age of 23 years, and the UC had been in total recession for the past 20 years. Upon admission, the patient’s initial laboratory investigation showed normal renal function, an increased white blood count, elevated C-reactive protein, and stools with blood.

The patient received antibiotics and steroids, and the symptoms improved. On the third hospitalization day, he complained of pain in the lower abdomen, and oliguria was recorded by the medical staff. The urine output did not improve after fluid administration, and the oliguria deteriorated rapidly to anuria. In addition, the blood examinations on the third day showed a sudden increase in both the creatinine and urea levels (Table 1).

**Table 1:** Laboratory results of the patient obtained on admission day one, preoperatively on day three, and postoperatively on day six WBC: white blood cell; CRP: C-reactive protein

Laboratory/ Hospital days	WBC (3.90-11.10 K/μL)	Urea (10-50mg/dl)	Creatinine (0.50-1.10mg/dl)	CRP (<0.5 mg/dl)	Urine pH (4.5 – 8)	Urine specific gravity (1.005-1.030 mg/ml)	Daily urine output (mL)
Day 1	20.20	13	0.59	1.62	6.0	1.035	1500
Day 3	16.50	11	1.97	2.85	6.0	1.030	230
Day 6	12.40	9	0.45	1.38	7.0	1.012	1650

**Figure 1:**
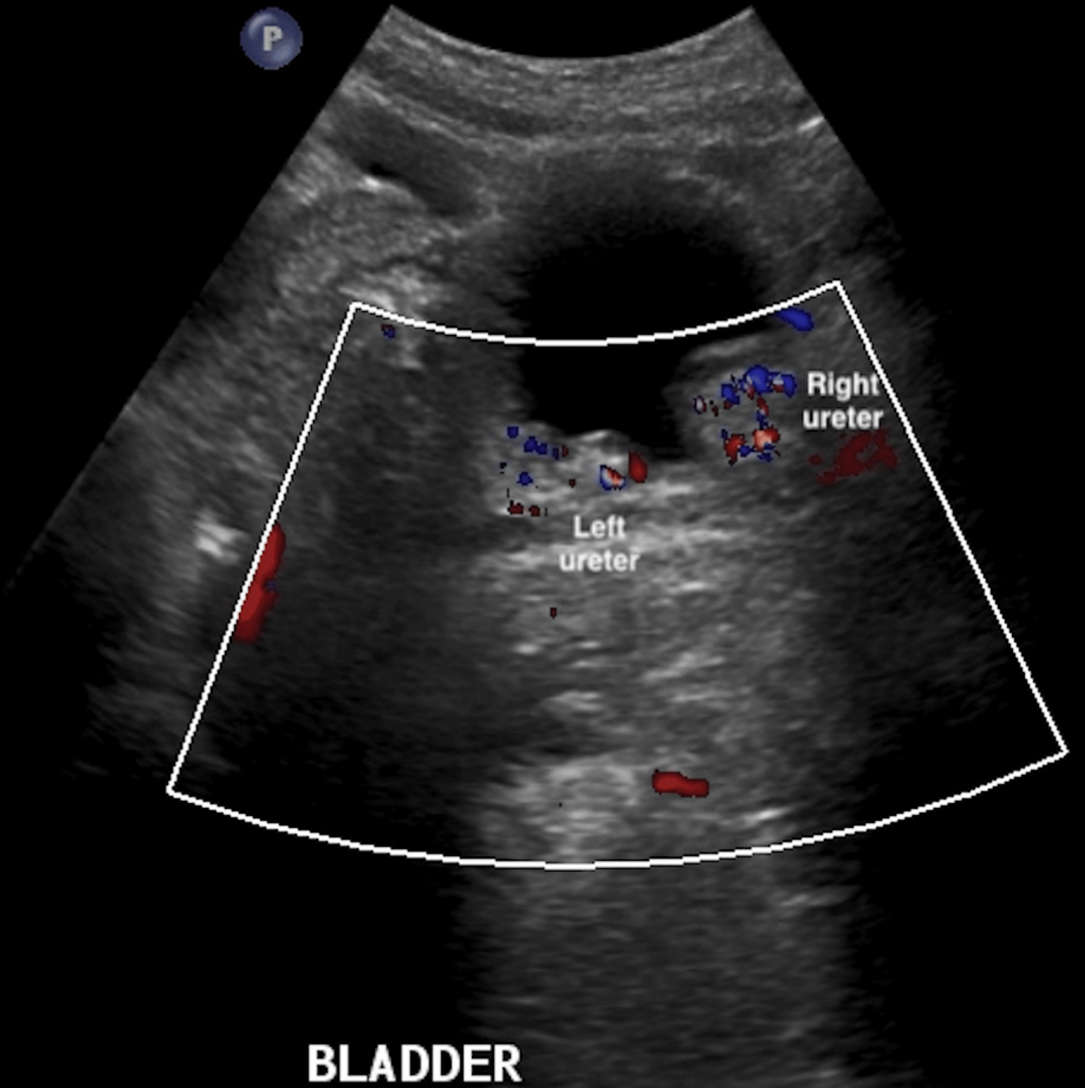
The ultrasound revealed bilateral ureterovesical junction obstruction, with both ureters contrasting at the lower level when applying the Doppler tool.

The Doppler ultrasound revealed bilateral UVJ obstruction, with both ureters contrasting at the lower level and dilated proximally (Figure 1).

**Figure 2:**
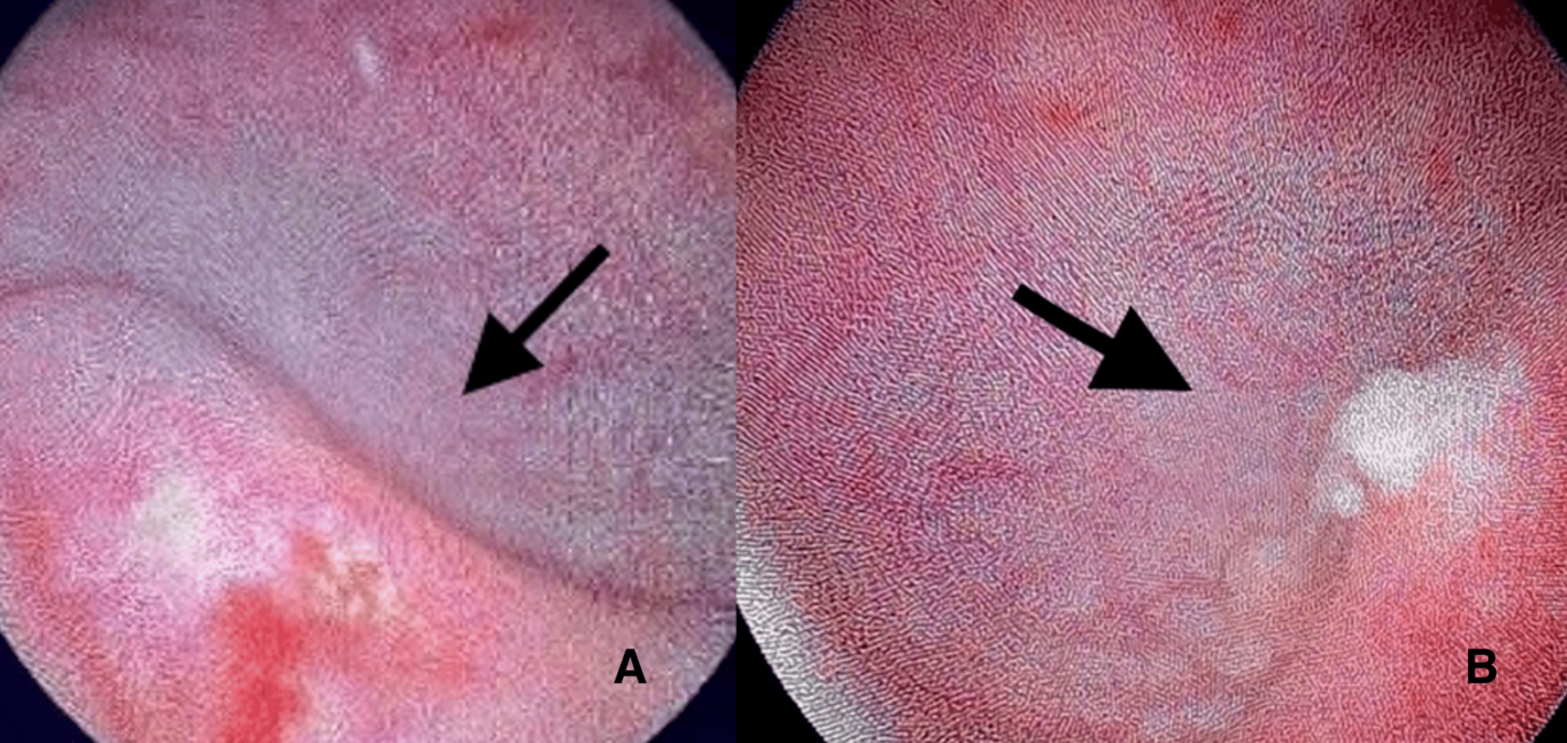
Cystoscopy view of the right ureter orifice (A) and the left ureter orifice (B); They are both edematous and obstructed by calculi. The endoscopic removal of the calculi followed.

A cystoscopy was performed promptly, and thin, fragile calculi obstructing both edematous ureteral orifices were observed (Figure 2).

The calculi were removed endoscopically, and a laborious placement of a double-J catheter in each ureter was followed to prevent renal deterioration. Postoperatively, the ultrasound showed significant improvement in the dilated ureters, and the blood tests revealed normalization of the serum creatinine and urea levels. The patient had normal urine output and was maintained on steroids, antibiotics, and mesalazine with no clinical relapse of UC. He was discharged home with a prescription of prophylactic antibiotics (daily dose-oral intake) and a scheduled admission after three weeks in the pediatric surgery department for the removal of the double-J catheters. On follow-up, the patient had normal ultrasound findings and no clinical signs of urolithiasis recurrence. 

## Discussion

Inflammatory bowel disease is a chronic, inflammatory disorder with two subtypes: CD and UC. Inflammatory bowel disease is considered a heritable, immune-mediated condition that develops as a result of the interaction between environmental factors, the mucosal response of the host, and the intestinal microbiota in a genetically predisposed host. Approximately a pediatric onset of disease occurs in about 10%-25% of the cases, while the rest of them occur during early adulthood [3, 4]. Pediatric UC usually presents with pancolitis rather than left-sided colitis, and gastrointestinal symptoms include diarrhea, rectal bleeding, abdominal pain, and poor growth [4]. The prevalence of extraintestinal manifestations (EIMs) is similar between adults and children and varies from 6% to 46%. Almost every organ can be involved; however, the most common extraintestinal symptoms affect the skin, the joints, the eyes, and the biliary tract [5, 6]. Quite less described and less frequent, EIMs may also affect the lungs, the heart, the vascular system, and the urinary system [5]. The family history of IBD and the early, pediatric disease onset, as presented in our patient, are both predisposing factors, probably indicating either a common pathophysiological path or autoimmune susceptibility, and less likely the extraintestinal manifestations as secondary results of gastrointestinal disease [6]. Concerning renal diseases in pediatric and adult patients with IBD, the differential diagnoses frequently include nephrolithiasis, tubulointerstitial nephritis, glomerulonephritis, and amyloidosis [5]. Renal stone presentation in the context of adult IBD is a well-reported entity in the literature, and the prevalence of nephrolithiasis among IBD adults is considered higher than the general population and varies from 12% to 28% [5, 7]. However, pediatric cases of urolithiasis in IBD are quite rare, as they account for only 10% of those in adults. Urolithiasis is estimated at 0.37% to 1% in the context of pediatric IBD and is often underdiagnosed [8]. It is more often associated with CD children rather than UC patients, especially those who underwent bowel surgery in the context of postoperative malabsorption [7]. What is more, although the UVJ is a common ureter obstruction location in children, the obstruction typically occurs in the ipsilateral ureter [9]. Bilateral proximal ureteral and UVJ obstruction is extremely rare and, to our knowledge, has not been reported in the context of a pediatric UC patient.

Interestingly, our patient had bilateral ureteral obstruction in the context of ulcerative colitis and had not undergone any kind of bowel surgery, becoming, that way, an exceedingly rare clinical case. We applied blood serum examinations, urine tests, a urine culture, and ultrasounds of the urinary system to define a probable pathophysiological mechanism. Considering the general and non-IBD-related causes of nephrocalcinosis, the patient had a clear personal and family history, and all the examinations of blood and urine samples did not indicate any kind of non-IBD-related factors (Table 1). Hence, the literature research was focused on the IBD-related causes of urolithiasis. While reviewing the literature for a suggestive pathophysiological mechanism that resulted in this urgent condition for the boy, many probable explanations were found, some of them compatible with our patient’s characteristics. To begin with, dehydration secondary to diarrhea or low liquid intake may contribute to stone formation [8]. Furthermore, the intestinal malabsorption of bile acid and fatty acids results in urine supersaturation of calcium oxalate and the formation of calcium oxalate stones [8]. This condition was not present in our patient, as it affects mainly CD patients with terminal ileum disease or patients with a bowel-surgery history [8]. Another risk factor of urolithiasis is hypercalciuria [10], although not established in our patient’s laboratory results. Considering other urine, biochemical factors contributing to stone formation, hypomagnesuria and hyperoxaluria are described in the literature [10], as well as the presence of a urinary infection, which is an important lithogenic cause [7]. Regarding these causes, the patient's urine laboratory tests did not correspond and were negative for infection. Last but not least, the medications should be highlighted, as they are claimed to be responsible for 1% of the urolithiasis cases in IBD patients [6, 8]. Corticosteroids, many antibiotics, such as ceftriaxone, sulfasalazine, mesalazine, and the antitumor necrosis factor-alpha agents (anti-TNFα) have been associated with lithogenic activity and consequences [7, 8]. Although our patient did not totally coordinate with the majority of the above-mentioned risk factors, it should be highlighted that he had diarrhea a few days before the bilateral obstruction occurred and he had been receiving mesalazine and the anti-TNFα agent infliximab for a few months. There is no literature data describing the duration of the medication and the lithogenic risk, so we carefully mention this condition. 

Putting the pieces together, the bilateral lithiasis in the context of pediatric UC is extremely rare. The increased clinical suspicion in the diagnostic process of sudden oliguria or anuria seems to result in a prompt and safe diagnosis and management. 

## Conclusions

Although urinary tract manifestations are described in IBD, bilateral ureteral obstruction is extremely rare. To the best of our knowledge, this is the first report of a bilateral UVJ obstruction, requiring endoscopic removal of the calculi in such a patient. Sudden deteriorating oliguria in the context of UC, may indicate secondary obstructive uropathy. The close monitoring of the patient's laboratory examinations, as well as the urine output and the liquid balance, can be very helpful with the differential diagnosis. Along with a meticulous Doppler ultrasound, they can all lead to prompt and correct identification of the clinical entity. Early diagnosis brings immediate intervention that should be applied to prevent acute kidney injury.

## References

[1] Yu YR, Rodriguez JR (2017). Clinical presentation of Crohn's, ulcerative colitis, and indeterminate colitis: symptoms, extraintestinal manifestations, and disease phenotypes. Semin Pediatr Surg.

[2] Khan R, Kuenzig ME, Benchimol EI (2023). Epidemiology of pediatric inflammatory bowel disease. Gastroenterol Clin North Am.

[3] Bouhuys M, Lexmond WS, van Rheenen PF (2023). Pediatric inflammatory bowel disease. Pediatrics.

[4] Sauer CG, Kugathasan S (2009). Pediatric inflammatory bowel disease: highlighting pediatric differences in IBD. Gastroenterol Clin North Am.

[5] van Hoeve K, Hoffman I (2022). Renal manifestations in inflammatory bowel disease: a systematic review. J Gastroenterol.

[6] Corica D, Romano C (2016). Renal involvement in inflammatory bowel diseases. J Crohns Colitis.

[7] Kumar S, Pollok R, Goldsmith D (2023). Renal and urological disorders associated with inflammatory bowel disease. Inflamm Bowel Dis.

[8] Bianchi L, Gaiani F, Bizzarri B (2018). Renal lithiasis and inflammatory bowel diseases, an update on pediatric population. Acta Biomed.

[9] Reich DA, Bayne CE, Sharadin CA, DeMarco RT (2023). Bilateral proximal ureteral and ureterovesical junction obstruction in a child. Urol Case Rep.

[10] McConnell N, Campbell S, Gillanders I, Rolton H, Danesh B (2002). Risk factors for developing renal stones in inflammatory bowel disease. BJU Int.

